# Aetiology and antibiotic susceptibility of bacterial keratitis at a referral centre in southern Sweden

**DOI:** 10.1038/s41598-025-04404-7

**Published:** 2025-06-20

**Authors:** Elin Österhed, Karl Oldberg, Ingemar Gustafsson

**Affiliations:** 1 Department of Clinical Sciences, Lund University, Lund, Sweden; 2 Department of Ophthalmology, Skåne University Hospital, Lund, Sweden; 3 Clinical Microbiology, Infection Control and Prevention, Office for Medical Services, Region Skåne, Lund, Sweden

**Keywords:** Bacterial keratitis, Keratitis aetiology, Keratitis treatment, Antibiotic susceptibility, Ophthalmology, Corneal diseases, Clinical microbiology

## Abstract

This was a retrospective cross-sectional study evaluating the aetiology and antibiotic susceptibility in patients treated for suspected bacterial keratitis at Skåne University Hospital during 2019. Inclusion criteria: eyes with bacterial keratitis. Exclusion criteria: co-infection with other microbes. Primary outcome parameters: predisposing factors, causative pathogens and antibiotic susceptibility. Secondary outcome parameter: antibiotic treatment. A total of 255 cases met the inclusion criteria. Of these, 149 (58%) occurred in contact lens wearers. Corneal cultures, when performed, were positive in 51% of cases. For eyes which had received antibiotic treatment prior to corneal culture (n = 36), the proportion of positive cultures was 50%. Ulcers < 1 mm were less likely to yield a positive culture than those ≥ 1 mm. The most frequently isolated bacteria were coagulase-negative staphylococci (48%). Antibiotic resistance rates were lowest to levofloxacin (0%), ciprofloxacin (2%) and chloramphenicol (4%), and highest to fusidic acid (47%) and clindamycin (19%). The low proportion of positive cultures from small ulcers suggests that these warrant a different diagnostic approach. Furthermore, corneal cultures from eyes with ongoing antibiotic treatment were positive to the same extent as those from untreated eyes, suggesting that discontinuation of antibiotic treatment before re-culturing might not be necessary.

**Supplementary Information:**

The online version contains supplementary material available at 10.1038/s41598-025-04404-7.

## Introduction

Bacterial keratitis is a sight-threatening infection of the cornea which requires urgent initiation of adequate treatment^1^. Except for a few pathogens, e.g. *Neisseria gonorrhoeae*, which can penetrate intact corneal epithelium, the healthy epithelium does not typically allow bacterial infiltration^2,3^. Consequently, bacterial keratitis is usually preceded by a disruption of the corneal epithelium due to trauma, ocular surgery, ocular surface disease or inflammatory conditions^2,4^. However, the use of contact lenses is a major predisposing factor for bacterial keratitis; the risk being especially high when contact lenses are worn overnight^5–7^.

In high-income countries, bacteria account for most cases of non-viral microbial keratitis, and Gram-positive species are more common than Gram-negative^8,9^. Some species, such as *Pseudomonas aeruginosa and Streptococcus pneumoniae*, have been associated with poorer clinical outcome^1,10^. From the Swedish perspective, a study conducted in Örebro County during 2004–2014 reported that 45% of culture-positive cases of infectious keratitis were in contact lens wearers, while a recent study in another region, Östergötland, found the most common predisposing factors to be contact lens wear and severely ill/blind eye (26% and 24% of isolates, respectively)^11,12^. In both these studies, the vast majority of positive cultures were purely bacterial, the most commonly isolated species being coagulase-negative staphylococci (CoNS).

Although keratitis is diagnosed using slit lamp biomicroscopy, corneal smear with Gram staining and culture should be considered in all cases to determine the causative bacteria, and for differentiation from fungal keratitis^13,14^. The recommendation in Sweden is to culture all cases, while the American Academy of Ophthalmology recommends smears and cultures in certain circumstances, for example, a large (> 2 mm) or central corneal infiltrate^13,15^.

The recommended initial treatment for bacterial keratitis is topical antibiotic eye drops^13,15^. Both monotherapy with a topical fluoroquinolone and combination therapy with fortified antibiotic eye drops, usually an aminoglycoside or vancomycin combined with a cephalosporin, have been shown to be efficient empirical treatments^13,16^. Antibiotic ophthalmic ointments are also available but, to the best of our knowledge, no randomized controlled trials comparing the efficacy of antibiotic ointments to that of eye drops in bacterial keratitis have been reported, and ointments are currently only recommended as a complement to drops^13^. Adjunctive therapy with topical corticosteroids has not been proven to improve visual outcome, although there is some evidence that it might be beneficial in certain subgroups^17^. Despite adequate treatment, bacterial keratitis may lead to significant visual loss due to corneal scarring and, in severe cases, corneal perforation and endophthalmitis, complications that sometimes warrant evisceration^1^.

Some studies have reported an increasing resistance to fluoroquinolones among bacteria isolated in bacterial keratitis globally^13,18–20^. However, it was found in a systematic review of data from the USA that the most recent studies showed a reduced or unchanged antibiotic resistance among ocular bacterial pathogens^21^. In the Swedish studies from Örebro and Östergötland counties the overall antibiotic resistance was found to be low, although about half of the *S. pneumoniae* isolates showed reduced susceptibility to fluoroquinolones^11,12^.

The purpose of this study was to examine the aetiology, antibiotic susceptibility and treatment administered to patients for suspected bacterial keratitis at a referral centre in southern Sweden. Since causative pathogens in bacterial keratitis and antibiotic susceptibility patterns show a geographical variation and may change over time, recent and local data are relevant to ensure that patients are given appropriate antibiotic treatment^8,9^.

## Subjects and method

This study was conducted as a retrospective cross-sectional evaluation of all cases of suspected bacterial keratitis treated at the Department of Ophthalmology at Skåne University Hospital, at Lund and Malmö, in southern Sweden. The study was performed in accordance with the Declaration of Helsinki and the General Data Protection Regulation (GDPR) of the European Union. Ethical approval was obtained from the Swedish Ethical Review Authority, no. 2023-04646-01. The need for informed consent from participants was waived by the Swedish Ethical Review Authority.

Patients assigned International Classification of Diseases 10th revision (ICD-10) code H16.9 during 2019 were identified in the local patient registry at Skåne University Hospital. Data were obtained from medical records and from the Department of Clinical Microbiology.

### Inclusion and exclusion criteria

Eyes deemed to have an active episode of bacterial keratitis during 2019 were included. In patients with more than one episode of bacterial keratitis during the study period, each eye and episode were included as separate cases. Eyes were included regardless of the localisation of the corneal ulcer. Eyes with a fungal, parasitic or viral co-infection, either suspected or verified by culture or PCR, as well as eyes in which the aetiology remained uncertain on the last visit, were excluded. The aetiology was assessed by a resident in ophthalmology, with co-assessment by a senior consultant in ophthalmology in selected cases. Cases not mainly treated at Skåne University Hospital were also excluded, i.e., patients whose first visit was to our clinic, but who were subsequently treated at a different clinic. These were patients from nearby cities without 24-h ophthalmological services who presented during evenings and weekends, or who were visiting the area temporarily. Patients referred by other ophthalmological clinics who were subsequently treated at Skåne University Hospital were included.

The size of the corneal epithelial defect, the choice of antibiotic and other treatments, adverse effects and the result of cultures from corneal scraping, including antibiotic susceptibility, were collected from the patients’ medical records.

### Corneal cultures

Corneal cultures were performed at the discretion of the treating ophthalmologist. All corneal cultures at our clinic are assessed for both bacterial and fungal growth, while direct microscopy, cultures or PCR for *Acanthamoeba*, and PCR for viruses are not routinely performed in cases of suspected bacterial keratitis. Our clinic’s local guidelines for corneal cultures state that material should be collected from the corneal ulcer using a sterile cotton-tipped applicator and sterile steel blades, and inoculated onto three different agar plates (chocolate agar, blood agar and Sabouraud agar). Subsequently, a sterile blade with collected material is transferred to a test tube containing Fastidious Anaerobe Broth. Plates were incubated for four days, blood agar and chocolate agar in 5% CO_2_ at 35 °C, and Sabouraud agar in air at 30 °C. Fastidious Anaerobe Broth cultures were incubated anaerobically for three days, and subsequently inoculated onto blood agar, chocolate agar and Fastidious Anaerobe agar for continued incubation for a further four days. Bacterial growth on the inoculated site on the original agar plates or in the broth was always reported, and species were identified with MALDI-TOF MS (Bruker Daltronics, Bremen, Germany). Antimicrobial susceptibility testing was performed with the EUCAST (European Committee on Antimicrobial Susceptibility Testing) disk diffusion method or with gradient strips, according to the recommendations of the manufacturers. Antibiotic susceptibility was classified as *S* (susceptible at normal dosing), *I* (susceptible at increased exposure), or *R* (resistant), based on the EUCAST clinical breakpoints, versions 8.1 and 9.0^22–24^. The breakpoints for *Moraxella catarrhalis* were used for all *Moraxella* species. For isolates without clinical breakpoints, only the minimum inhibitory concentration (MIC) was reported.

### Statistical methods and calculations

Microsoft® Excel® for Microsoft 365 MSO (Version 2408 Build 16.0.17928.20114) 32-bit was used for descriptive statistics (Microsoft Corporation, Redmond, Washington, USA). Comparative statistical analyses were performed with IBM SPSS for Windows, version 28.0.1.0 (IBM Corp, Armonk, New York, USA) using Fisher’s Exact Test. A *p*-value ≤ 0.05 was considered significant.

The primary outcome parameters were predisposing factors, causative pathogens and antibiotic susceptibility patterns of isolated bacteria. The secondary outcome parameter was the antibiotic treatment.

## Results

**Fig. 1:**
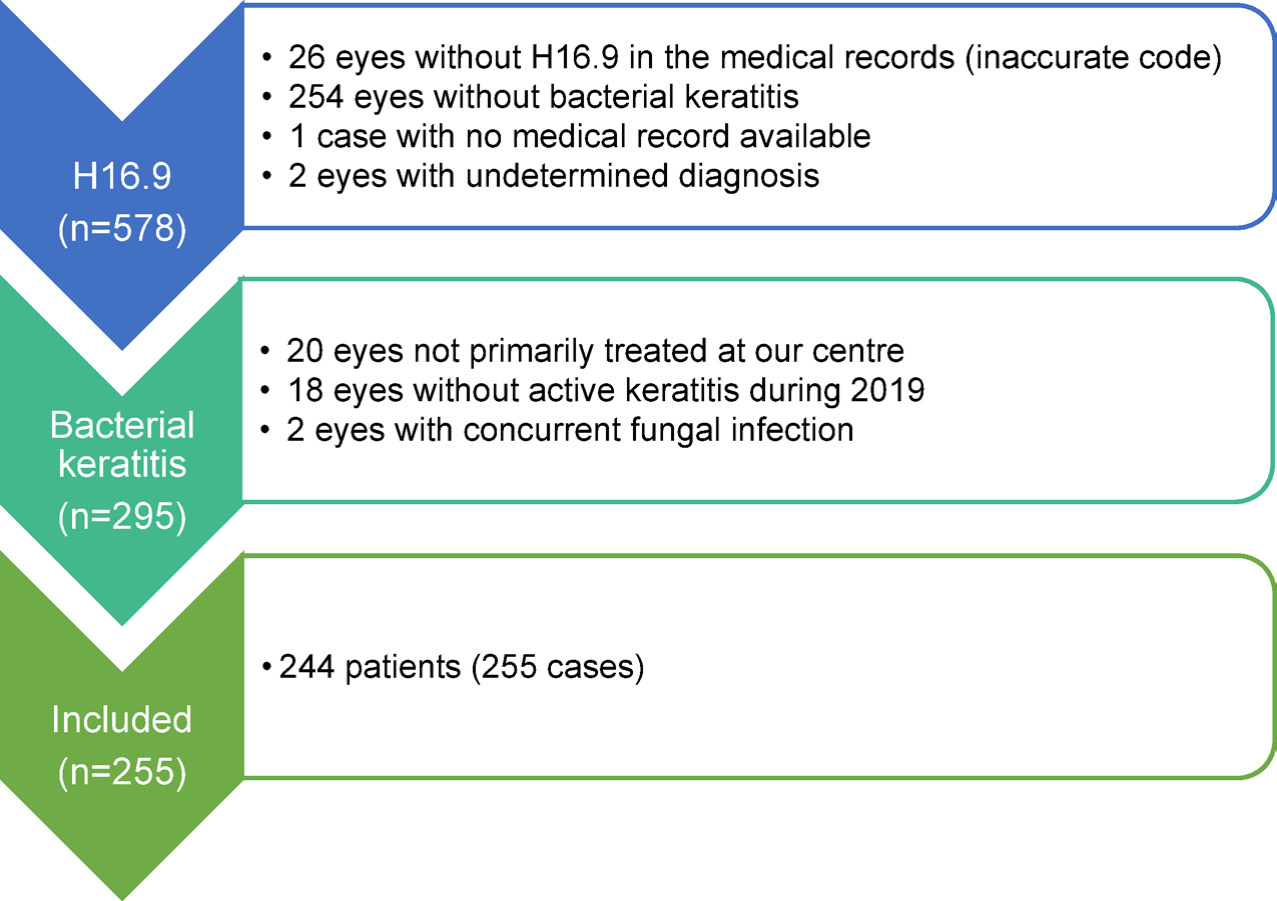
Included cases. Flow chart showing inclusion in and exclusion from the present study.

A total of 578 patients with ICD-10 code H16.9 were identified during 2019. After review of the patients’ medical records, 295 eyes were deemed to have bacterial keratitis. Out of these, 255 eyes met the inclusion criteria (Fig. 1).

### Predisposing factors

The most common predisposing factor was contact lens wear (58%), followed by trauma (9%), inflammation such as blepharitis and atopic keratoconjunctivitis (6%) and ocular surgery (4%). Other predisposing factors were present in 13% of cases and included mainly severe glaucoma, ectropion, entropion, lagophthalmos and reduced corneal sensitivity. In 10% of cases, no predisposing factor was identified.

### Corneal cultures

Corneal cultures were performed in 82% (n = 208) of the eyes, 51% (n = 107) of which were positive, and resulted in 147 individual bacterial isolates. More than one bacterial strain was isolated in 29% (n = 31) of positive cultures. The majority of cultures were performed according to the local guidelines, but 7% (n = 15) were performed using a swab only. The reason for not performing a corneal culture was known in 11 of 47 cases, and were prior antibiotic treatment (n = 5), lesion size deemed to small (n = 3), inability to collect samples due to patient factors (n = 1), and incorrect sample handling (n = 2).

Antibiotics had been administered to 17% (n = 36) of the 208 eyes before the cultures were made. Of these, 50% (n = 18) were positive (Table 1), compared with 51% (n = 86) of eyes not treated with antibiotics prior to culture. In three cases, it was unknown if antibiotic treatment had been initiated prior to culture. The most common antibiotic therapy initiated before corneal culture was chloramphenicol in monotherapy (20/36). Details on antibiotic treatment administered prior to corneal culture are available in Supplementary Table S1 online.

**Table 1:** Culture results in cases receiving prior antibiotic treatment.

	< 1 mm, n (%) n = 8	1–2 mm, n (%) n = 7	> 2 mm, n (%) n = 10	All cases, n (%) n = 36
Positive	3 (38)	5 (71)	5 (50)	18 (50)
Negative	5 (63)	2 (29)	5 (50)	18 (50)

Culture results in cases receiving antibiotic treatment prior to corneal cultures. The epithelial defect size was known in 25 of the 36 cases.

**Fig. 2:**
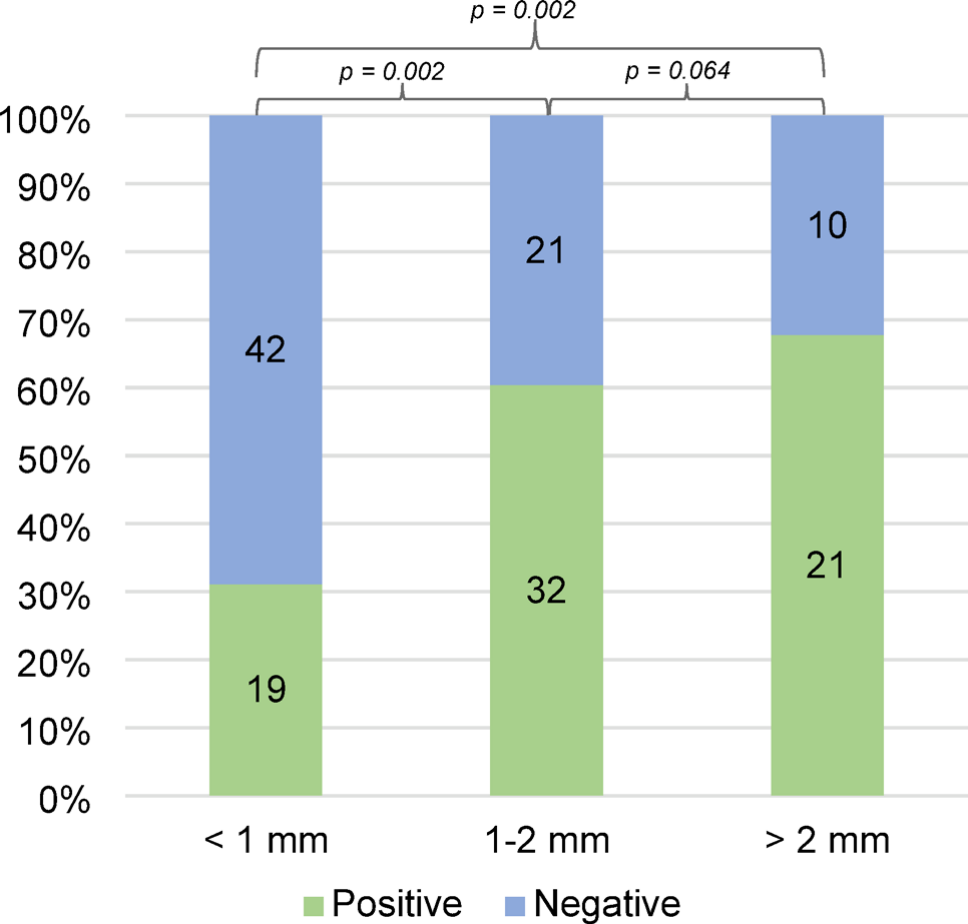
Culture results according to epithelial defect size. Culture results according to the size of the epithelial defect on presentation. Only cultured cases in which the size of the epithelial defect was known are included. The number of cases are shown inside the stacks.

The size of the epithelial defect was known in 70% (n = 145) of cultured cases. Of these, 42% (n = 61) were < 1 mm, 37% (n = 53) 1–2 mm, and 21% (n = 31) > 2 mm. Corneal ulcers with an epithelial defect < 1 mm were less likely to yield a positive corneal culture (Fig. 2).

Gram-positive and Gram-negative bacteria constituted 82% and 18% of isolates, respectively. The most frequently isolated bacterial strains are shown in Table 2. *S. epidermidis* accounted for 76% (n = 53) of CoNS isolates. Culture verified *P. aeruginosa* keratitis was more common in contact lens wearers than in non-contact lens wearers, *p* = 0.047, while all isolates of *Moraxella spp.* came from non-contact lens wearers. Table 3 shows isolated bacterial strains according to the size of the epithelial defect.

**Table 2:** Isolated species.

	Non-CLW, n (%) n = 88	CLW, n (%) n = 120	*p*-value	All cultured cases, % n = 208	All isolates, % n = 147
CoNS, n = 70	31 (35.2)	39 (32.5)	0.767	33.7	47.6
*Corynebacterium* spp, n = 14	8 (9.1)	6 (5.0)	0.273	6.7	9.5
*S. aureus*, n = 13	9 (10.2)	4 (3.3)	0.078	6.3	8.8
*C. acnes*, n = 6	1 (1.1)	5 (4.2)	0.405	2.9	4.1
*S. pneumoniae*, n = 6	5 (5.7)	1 (0.8)	0.085	2.9	4.1
Other *streptococci*, n = 5	5 (5.7)	0 (0.0)	0.013	2.4	3.4
*P. aeruginosa*, n = 13	2 (2.3)	11 (9.2)	0.047	6.3	8.9
*Moraxella* spp, n = 9	9 (10.2)	0 (0.0)	< 0.001	4.3	6.1
Other species, n = 11	9 (10.2)	2 (1.7)	0.010	5.3	7.5
Culture positive cases	54 (61.4)	53 (44.2)	–	107 (51.4)	

Isolated bacterial species in all cases and comparison of isolated bacterial species in non-contact lens wearers and contact lens wearers. Only cultured cases are included in this analysis, n = 208. *C. acnes*, *Cutibacterium acnes*; CoNS, coagulase-negative staphylococci; *P. aeruginosa*, *Pseudomonas aeruginosa*; *S. aureus*; *Staphylococcus aureus*; *S. pneumoniae*, *Streptococcus pneumoniae*; spp, species.

**Table 3:** Bacterium according to size of epithelial defect.

	< 1 mm (%) n = 22	1–2 mm (%) n = 44	> 2 mm (%) n = 35
Skin flora, n = 62	20 (91)	27 (61)	15 (43)
*Moraxella* spp, n = 6	1 (5)	2 (5)	3 (9)
*S. aureus*, n = 8	1 (5)	5 (11)	2 (6)
*P. aeruginosa*, n = 10	0 (0)	6 (14)	4 (11)
*S. pneumoniae*, n = 4	0 (0)	1 (2)	3 (9)
Other species, n = 11	0 (0)	3 (7)	8 (23)

Isolated bacteria according to the size of the epithelial defect on presentation. Only bacteria isolated from eyes in which the epithelial defect size was known are included, n = 101. Skin flora includes coagulase-negative staphylococci, *Cutibacterium acnes* and *Corynebacterium* species. Spp, species; *S. aureus*, *Staphylococcus aureus*; *P. aeruginosa*, *Pseudomonas aeruginosa*; *S. pneumoniae*, *Streptococcus pneumoniae.*

### Antibiotic susceptibility

**Fig. 3:**
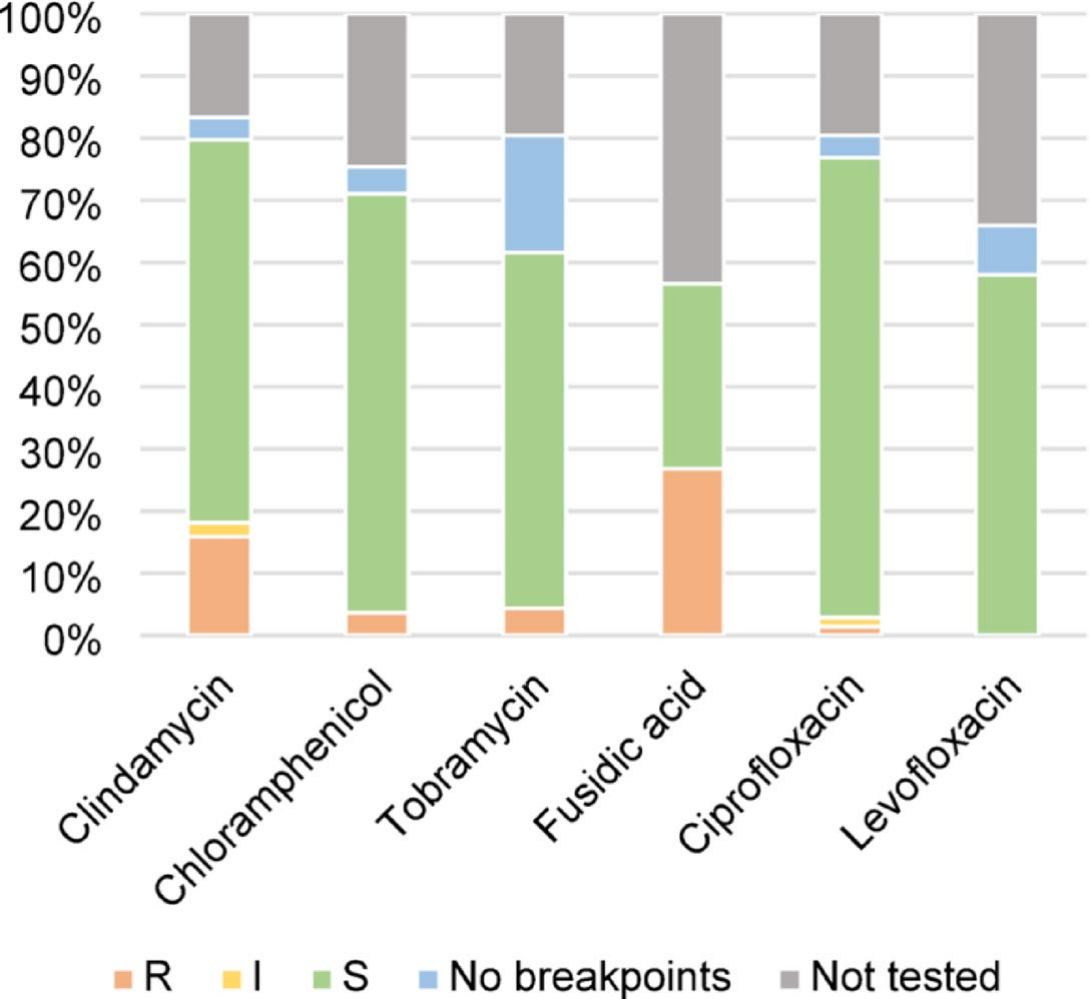
Antibiotic susceptibility. Antibiotic susceptibility for available topical antibiotic agents for 138 isolates. No antibiotic susceptibility testing was performed on 9 isolates, these have been excluded in this diagram. If there were no clinical breakpoints, only the MIC (minimal inhibitory concentration) was reported (shown as ”No breakpoints”). Details can be found in supplemental information. MIC ranges (mg/L): Clindamycin 0.032–4.0; Chloramphenicol 0.064–8.0; Tobramycin 0.032–32.0; Ciprofloxacin 0.008–0.25; Levofloxacin 0.032–1.0. R, resistant; I, susceptible at increased exposure; S, susceptible.

Testing for antibiotic susceptibility was performed on 94% (n = 138) of the isolates. Antibiotic susceptibility for all isolates is shown in Fig. 3, with details available in Supplementary Table S2 online. All of the 13 *P. aeruginosa* isolates were susceptible to tobramycin and ciprofloxacin. Disk diffusion zone diameter for ciprofloxacin was available for 11 *P. aeruginosa* isolates, and classified all as wildtype for ciprofloxacin, i.e. without acquired resistance. Antibiotic susceptibility to levofloxacin was tested in two of 13 *P. aeruginosa* isolates, and both were susceptible. All six *S. pneumoniae* isolates were sensitive to clindamycin and chloramphenicol. Three *S. pneumoniae* isolates were tested for susceptibility to levofloxacin and were susceptible, while none were tested for susceptibility to ciprofloxacin since there are no clinical breakpoints. No strains of methicillin-resistant *S. aureus* were found.

### Antibiotic treatment

The most common antibiotic agents used for empirical treatment were tobramycin (78%), levofloxacin (59%), clindamycin (31%) and chloramphenicol (24%). Combination therapy with two antibiotic agents were used in 87% of cases, the most frequently used regimen being levofloxacin drops with tobramycin depot drops (40%). Three antibiotic agents were used in 10%, while monotherapy was used in 3% of cases. Antibiotic regimens used in more than 2 cases are shown in Supplementary Fig. S1 online. Side effects of antibiotic treatment were suspected in 13% (n = 33) of cases, of which 31 were ocular discomfort on installation of the antibiotic agent and punctate epithelial defects at follow-up, and two were deemed to be allergic reactions, one local and one systemic (dyspnoea).

## Discussion

The results of this study show that contact lens wear was the most common cause of bacterial keratitis (58%), followed by ocular trauma (9%). Several other studies in developed countries have shown contact lens wear to be the most important risk factor for microbial keratitis (22–63%)^11,12,25–30^. Ocular surface disease or blepharitis has been reported to account for 18–23% of cases, which was not seen in the present study^25–30^. However, this may be explained by the way in which predisposing factors were classified or a lack of documentation of blepharitis and ocular surface disease in the medical records in the current study.

Corneal cultures were positive in 51% of the cultures. Previous studies report varying rates of positive corneal cultures in patients with bacterial keratitis, ranging from 50 to 86%^18,31,32^. The fact that 7% of cultures were not taken according to the recommendations might affect the corneal culture positivity rate. Interestingly, our results show that ulcers with an epithelial defect smaller than 1 mm were statistically less likely to yield a positive culture (31%) than lesions of 1–2 mm (60%) and > 2 mm (68%). This could possibly be explained by a lower volume of infected tissue resulting in a lower sensitivity. Smaller ulcers have been shown to have a significantly better clinical outcome and heal faster^30^. These factors may suggest that cultures are of limited importance in small ulcers, however, sampling these smaller ulcers may involve removing the source of infection, which could promote healing. The fact that 42% of lesions were < 1 mm could contribute to the lower culture positivity rate. However, this might not fully explain the lower culture positivity rate, since the study by Schaefer et. al., in which the culture positivity rate was 86%, had a similar rate of lesions > 2 mm (23.5%)^31^.

Moreover, we found that 50% of the cultures from eyes that had been treated with antibiotics prior to culturing were positive, implying that corneal cultures are indicated even when antibiotic therapy has already been initiated. It could be suspected that smaller lesions would be more prone to yield negative cultures after treatment than larger ones, but the number of such cases with a known epithelial defect size was too small to allow for subgroup analysis. The type of antibiotic administered likely affects the chance of isolating the causative pathogen, and 26 of the 36 patients had received antibiotic regimens which are not recommended for bacterial keratitis. The dosage and duration of antibiotic therapy prior to corneal culture are likely also of importance, but since these cases were referred to us from other health care givers, this information was unavailable, which constitutes a limitation. Nonetheless, the fact that prior antibiotic treatment did not affect corneal culture positivity rate supports performing a corneal culture without taking the risk of discontinuing antibiotic treatment. However, further studies are needed to better assess how prior antibiotic treatment affects culture results, especially in severe cases of bacterial keratitis which do not respond to recommended antibiotic therapy with adequate dosage.

In spite of the Swedish recommendations stating that a corneal culture should be performed in all cases of suspected bacterial keratitis, cultures were not obtained in 18% of cases in the present study. Due to the retrospective nature of this study, we lack data on the reason for not performing corneal cultures in a majority of these cases. However, there is indication that the most common reasons were prior antibiotic treatment and small epithelial defect size. It needs to be pointed out that this might affect the distribution of causative bacteria, and we have demonstrated that lesion size does indeed affect culture positivity rate. Based on the results of cultures obtained after antibiotic treatment, it is unlikely that the cases which were not cultured due to prior treatment significantly impact the overall culture positivity rate, but the effect on the distribution of isolated bacteria is uncertain.

The most frequently isolated bacteria in our study were CoNS, including *S. epidermidis*, as has been reported in several European studies^11,12,27,30^. Since skin flora (CoNS, *Cutibacterium acnes* and *Corynebacterium* species) are commonly found in conjunctival cultures from non-infected eyes, it can be debated whether they represent contamination or the causative agent^33^. On the one hand, the skin flora could be the causative agent, as the avascular cornea, compromised by an epithelial defect, could be more susceptible to infections by less pathogenic bacteria. Indeed, both *S. epidermidis* and *Corynebacterium* species have been reported to cause bacterial keratitis^34,35^. On the other hand, keratitis could be caused by another bacterial pathogen that evades detection due to the predominance of skin flora on the ocular surface. *P. aeruginosa* was more frequently isolated in contact lens wearers, and the prevalence among cultured cases was in line with findings of other recent Swedish studies^11,12^, although others found a higher prevalence^18,25,36^. In spite of contact lens wear being by far the most common predisposing factor, Gram-negative bacteria accounted for only 18% of isolates. One might expect a higher prevalence, since Gram-negative bacteria have been shown to be more frequently isolated in contact lens associated keratitis^5^. However, studies from Sweden and France have found similar prevalences of Gram-negative bacteria (16, 16 and 17%), although the study by Roth et. al. had a lower rate of contact lens associated cases (26%)^11,12,27^. The low prevalence of *P. aeruginosa* could explain the low rate of Gram-negative bacteria, although the reason for the low prevalence is uncertain.

The isolated bacteria showed the highest rate of antibiotic sensitivity to ciprofloxacin and chloramphenicol, suggesting that these are suitable for empiric treatment in our population. Antibiotic susceptibility testing was variable during the study period, resulting in a lack of data, most importantly for levofloxacin, for which antibiotic susceptibility was only tested in 91 of all 147 isolates, 2 of 13 *P. aeruginosa* isolates, and 3 of 6 *S. pneumoniae* isolates. Nonetheless, our results indicate that levofloxacin is also an adequate choice for initial treatment, since none of the isolates were resistant to it. Furthermore, all *P. aeruginosa* isolates tested for ciprofloxacin for which there were disk diffusion zone diameters were classified as wildtype for ciprofloxacin, i.e. without acquired resistance mechanisms. This makes resistance to levofloxacin less likely, since the major resistance mechanisms are the same for both agents^37^. In contrast, the highest resistance rates were seen for fusidic acid and clindamycin, making these less suitable for initial treatment. The reduced sensitivity of *S. pneumoniae* to fluoroquinolones found in the two recent Swedish studies^11,12^ was not confirmed in the present study, although testing of antibiotic susceptibility of *S. pneumoniae* to fluoroquinolones was limited. However, all *S. pneumoniae* isolates were susceptible to chloramphenicol.

Treatment patterns for bacterial keratitis vary significantly between different centres, although the types of antibiotics used are similar^27,31,38,39^. Monotherapy is infrequently used at our clinic, with only 3% of cases receiving initial monotherapy, compared to 41–84% as reported by others^28,30^. The Swedish recommendation is combination therapy with a fluoroquinolone and chloramphenicol^15^. Since previous studies have indicated resistance of *S. pneumoniae* to fluoroquinolones, the addition of a second antibiotic agent seems prudent and, based on our results, chloramphenicol is an adequate choice. According to Swedish guidelines, substitution of chloramphenicol with an aminoglycoside, e.g. tobramycin, should be considered if there is a high suspicion of *P. aeruginosa* keratitis, mainly in contact lens wearers^15^. While the present study supports treatment of *P. aeruginosa* keratitis with tobramycin, it also indicates that fluoroquinolones are appropriate for the initial treatment of contact lens wearers. However, further studies assessing the susceptibility of *P. aeruginosa* to levofloxacin are called for. This is of particular interest in our population, since levofloxacin was the most commonly used fluoroquinolone during the study period, and tobramycin eye drops are no longer commercially available in Sweden, but must be produced by compounding pharmacies, which is associated with higher costs and a shorter shelf life than commercially available drops^16,40^.

A strength of this study is the large number of consecutive cases at one centre. A limitation is that some data were missing from the medical records, e.g. predisposing factors and epithelial defect size. Also, mild side effects might have been underreported. As previously pointed out, the fact that the local recommendations for corneal cultures were not always followed, and inconsistent antibiotic susceptibility testing, constitutes the most important limitations of this study.

In conclusion, the predisposing factors and species isolated in cases of bacterial keratitis were consistent with the findings of previous studies. There was a considerable variation in resistance patterns, which must be carefully considered in clinical practice and when developing clinical guidelines. Based on the findings of this study, we have proposed appropriate treatment strategies for bacterial keratitis. Additionally, since eyes that had received antibiotic treatment prior to bacterial culturing were as likely to yield positive cultures as those that had not, discontinuing antibiotic treatment before collecting samples for new cultures may not be necessary, which is of considerable clinical relevance. Further studies assessing how different antibiotic regimens administered before corneal culture affect culture results could provide valuable information on how to best manage these patients, which is of certain importance in cases of severe keratitis, in which discontinuing antibiotic treatment constitutes a considerable risk. Furthermore, the low rate of positive cultures in small lesions constitutes a diagnostic challenge, and future studies assessing corneal culture results and clinical outcome in this specific subgroup, as well as evaluation of newer diagnostic methods, could aid in determining the best clinical approach to these cases.

## Electronic supplementary material

Below is the link to the electronic supplementary material.

Supplementary Material 1

## Data Availability

The datasets generated and analysed during the current study are available from the corresponding author on reasonable request.
