# Supplementary material for: Aetiology and antibiotic susceptibility of bacterial keratitis at a referral centre in southern Sweden
Source: Sci Rep. 2025 Jun 20;15:20123. doi: 10.1038/s41598-025-04404-7 (PMC12181293; doi:10.1038/s41598-025-04404-7)
Supplement: Supplementary file 1 — Supplementary Material 1 [file 41598_2025_4404_MOESM1_ESM.pdf]

Aetiology and antibiotic susceptibility of bacterial keratitis at a referral centre in southern Sweden

Elin Österhed*, Karl Oldberg, Ingemar Gustafsson

	Levofloxacin	Chloramphenicol	Tobramycin	Azitromycin	Fusidic acid	Polymyxin B	Clindamycin & Tobramycin	Chloramphenicol & Levofloxacin	Oral clindamycin & bibrocathol	Unspecified	Monotherapy	Double therapy
	n (%)	n (%)	n (%)	n (%)	n (%)	n (%)	n (%)	n (%)	n (%)	n (%)	n (%)	n (%)
**Positive, n=18**	3 (17)	9 (50)	0 (0)	1 (6)	2 (11)	1 (6)	1 (6)	0 (0)	0 (0)	1 (6)	16 (94)	1 (6)
**Negative, n=18**	3 (17)	11 (61)	1 (6)	0 (0)	0 (0)	1 (6)	0 (0)	1 (6)	1 (6)	0 (0)	16 (94)	1 (6)
**Total, n=36**	6 (17)	20 (56)	1 (3)	1 (3)	2 (6)	2 (6)	1 (3)	1 (3)	1 (3)	1 (3)	32 (94)	2 (6)

Supplementary Table S1 - Antibiotic treatment prior to corneal culture. Type of antibiotic treatment administered in the 36 cases receiving treatment proir to corneal culture. In one case the type of antibiotic treatment administered before culture was not specified, and in one case the patient had recieved oral clindamycin and topical bibrocathol, these two cases have beed excluded from the columns showing the cases which received mono- or double therapy.

	Clindamycin					Chloramphenicol					Tobramycin				
	S %	I %	R %	No CBP %	Tested n (%)	S %	I %	R %	No CBP %	Tested n (%)	S %	I %	R %	No CBP %	Tested n (%)
**CoNS n=70**	77	5	18	0	61 (87)	100	0	0	0	57 (81)	91	0	9	0	54 (77)
**Corynebacterium spp n=14**	69	0	31	0	13 (93)	0	0	0	100	3 (21)	0	0	0	100	9 (64)
**S aureus n=13**	100	0	0	0	12 (92)	92	0	8	0	13 (100)	100	0	0	0	13 (100)
**Cutibacterium acnes n=6**	100	0	0	0	4 (67)	100	0	0	0	3 (50)	0	0	0	100	4 (67)
**S pneumoniae n=6**	100	0	0	0	6 (100)	100	0	0	0	6 (100)	0	0	25	75	4 (67)
**Other streptococci n =5**	100	0	0	0	5 (100)	67	0	0	33	3 (60)	0	0	0	100	1 (20)
**Other G+ n=6**	40	0	0	60	5 (83)	50	0	0	50	2 (33)	0	0	0	100	4 (67)
**P aeruginosa n=13**	N/A	N/A	N/A	N/A	N/A	N/A	N/A	N/A	N/A	N/A	100	0	0	0	13 (100)
**Moraxella spp n=9**	N/A	N/A	N/A	N/A	N/A	100	0	0	0	9 (100)	0	0	0	100	4 (44)
**Other G- n=5**	0	0	100	0	2 (40)	75	0	0	25	4 (80)	80	0	0	20	5 (100)
**All isolates n=147**	73,9	2,6	19,1	4,3	115 (78)	89,4	0,0	4,8	5,8	104 (71)	71,2	0,0	5,4	23,4	111 (76)

	Fusidic acid					Ciprofloxacin					Levofloxacin				
	S %	I %	R %	No CBP %	Tested n (%)	S %	I %	R %	No CBP %	Tested n (%)	S %	I %	R %	No CBP %	Tested n (%)
**CoNS n=70**	48	0	52	0	61 (87)	100	0	0	0	60 (86)	100	0	0	0	62 (89)
**Corynebacterium spp n=14**	-	-	-	-	0(0)	100	0	0	0	13 (93)	0	0	0	100	6 (43)
**S aureus n=13**	100	0	0	0	12 (92)	88	13	0	0	8 (62)	100	0	0	0	11 (85)
**Cutibacterium acnes n=6**	-	-	-	-	0 (0)	-	-	-	-	0 (0)	0	0	0	100	1 (17)
**S pneumoniae n=6**	-	-	-	-	0 (0)	-	-	-	-	0 (0)	100	0	0	0	3 (50)
**Other streptococci n =5**	-	-	-	-	0 (0)	-	-	-	-	0 (0)	0	0	0	100	1 (20)
**Other G+ n=6**	-	-	-	-	0 (0)	0	0	0	100	4 (67)	0	0	0	100	3 (50)
**P aeruginosa n=13**	N/A	N/A	N/A	N/A	N/A	100	0	0	0	13 (100)	100	0	0	0	2 (15)
**Moraxella spp n=9**	-	-	-	-	0 (0)	75	0	25	0	8 (89)	100	0	0	0	2 (22)
**Other G- n=5**	0	0	100	0	2 (40)	60	20	0	20	5 (100)	-	-	-	-	0 (0)
**All isolates n=147**	52,6	0,0	47,4	0,0	78 (53)	91,9	1,8	1,8	4,5	111 (76)	87,9	0,0	0,0	12,1	91 (62)

Supplementary Table S2 - Antibiotic susceptibility. Antibiotic susceptibility for isolates tested for the specified antibiotic agent in percentages. N/A signifies that the antibiotic agent lacks activity against the particular bacterial species.

S=susceptible, I=susceptible at incresed exposure, R=resistant, No CBP= no clinical breakpoints, N/A= not applicable

9 of the 147 isolates (6/70 CoNS, 1/14 Corynebacterium spp, 2/6 Cutibacterium acnes) were not tested for susceptibility to any antibiotic agent.

CoNS = *Coagulase-negative staphylococci* , S aureus = *Staphylococcus aureus* , S pneumoniae = *Streptococcus pneumoniae* , P aeruginosa = *Pseudomonas aeruginosa* , spp = species

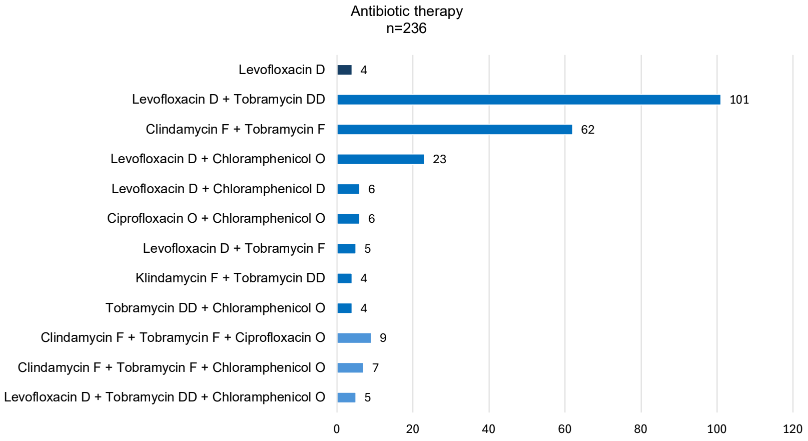

Supplementary Figure S1 – Antibiotic therapy. Antibiotic therapy initiated at the first visit in more than 2 cases, n=236 of 254 treated eyes.
Two of the excluded cases received intravitreal vancomycin and ceftazidime in combination with two different topical antibiotic agents.
D = drops, O = ointment, F = fortified drops, DD = depot drops
